# Formation of Hierarchical Porous Films with Breath-Figures Self-Assembly Performed on Oil-Lubricated Substrates

**DOI:** 10.3390/ma12183051

**Published:** 2019-09-19

**Authors:** Edward Bormashenko, Yelena Bormashenko, Mark Frenkel

**Affiliations:** Department of Chemical Engineering, Biotechnology and Materials, Engineering Sciences Faculty, Ariel University, Ariel 407000, Israel; yelenabo@ariel.ac.il (Y.B.); markfr@ariel.ac.il (M.F.)

**Keywords:** honeycomb polymer films, breath-figures self-assembly, oil-lubricated substrates, Voronoi entropy, Cassie wetting regime

## Abstract

Hierarchical honeycomb patterns were manufactured with breath-figures self-assembly by drop-casting on the silicone oil-lubricated glass substrates. Silicone oil promoted spreading of the polymer solution. The process was carried out with industrial grade polystyrene and polystyrene with molecular mass $M_{w}=35,000\frac{g}{mol}$. Both polymers gave rise to patterns, built of micro and nano-scaled pores. The typical diameter of the nanopores was established as 125 nm. The mechanism of the formation of hierarchical patterns was suggested. Ordering of the pores was quantified with the Voronoi tessellations and calculation of the Voronoi entropy. The Voronoi entropy for the large scale pattern was $S_{vor}=0.6-0.9$, evidencing the ordering of pores. Measurement of the apparent contact angles evidenced the Cassie-Baxter wetting regime of the porous films.

## 1. Introduction

There has been an increasing interest in micro-patterned surfaces in the last few decades due to their significant role in a variety of technologies, including biotechnology [1], tribology [2], optical [3] and microfluidics [4] applications. Micro-patterned surfaces enable the control of lining cells’ position, shape and function [1], constitution of dry and wet friction [2], design of the surfaces with prescribed optical properties [3] and smart manipulation of micro-volume liquids [4]. Manufacturing of micro-patterned surfaces is a key factor for industry implementation of biomimetic-inspired effects, such as the lotus- and shark skin-effects, allowing preparing superhydrophobic interfaces [5,6] and surfaces demonstrating low hydrodynamic drag [7]. A variety of advanced techniques have been implemented for manufacturing micro-patterned surfaces, including micro-printing [1], replica molding [3,8], photolithography, molecular assembly patterning, stencil-assisted patterning, ink-jet technology, laser-guided writing of patterns and exploiting surface instabilities [9,10,11]. 

One of the simplest and most effective methods enabling manufacturing micro-porous surfaces is the so-called breath-figures self-assembly method [12,13,14,15,16,17]. The breath-figures process is a commonly observed phenomenon in daily life. One example is the fog that appears on a window when we breathe on it [18]; this is also the origin of the term “breath-figure”. In the modern era, systematic study of the process of breath-figures water condensation was carried out by Aitken [19], Rayleigh [20] and Baker [21]. The interest in the breath-figures process was revived when it was demonstrated that water condensation occurring under evaporation of rapidly evaporated polymer solutions results in formation of well-ordered, micro-porous, honeycomb patterns [22,23,24,25,26]. These patterns demonstrated a potential for photonics [27,28,29], membranes [30] and biotechnology [31]. The state-of-the-art in the field has been reviewed recently in Reference [32]. A profound understanding of the breath-figures self-assembly has been a challenging task and it has not been attained until now. We demonstrate the possibility to manufacture hierarchical porous structures with the breath-figures self-assembly realized with oil-lubricated surfaces. The paper elucidates the role of substrate in the breath-figures self-assembly. The silicone-oil lubricated substrates used in the study provided de-pinning of triple line of polymer solutions; thus, promoting uniform spreading of solutions under the drop-casting process. The topography of the reported patterns contains ensembles of micro and nanopores.

## 2. Materials and Methods

### 2.1. Materials

Two kinds of polystyrene (PS) were used for the breath-figures self-assembly. The first was PS with a molecular mass of $M_{w}=35,000\frac{g}{mol}$ supplied by Sigma-Aldrich (Israel, Rehovot). The second was industrial PS 143E, supplied by BASF SE, with a density of $\rho =1.043\frac{g}{cm^{3}}$. Silicone oil (Poly (dimethylsiloxane, PDMS)) supplied by Sigma-Aldrich with average $M_{n}=580$ and density $\rho =0.93\frac{g}{cm^{3}}$ was used as a lubricator. Glass slides of 18 × 18 mm with a thickness of 150 µm were used as solid substrates. A mixture of Dichloromethane (CH_2_Cl_2_) and Chloroform (CHCl_3_) was used as a solvent. Chemical grade solvents were supplied by Bio-Lab ltd. (Israel, Ashkelon). A 4 wt % PS solution was prepared by dissolving the polymer in a mixture of chloroform (7.6 wt %) and dichloromethane (87.4 wt %). It was demonstrated in Reference [33] that the aforementioned mixture of chlorinated solvents promotes the formation of the breath-figure patterns.

### 2.2. Methods

#### 2.2.1. Preparation of Silicone Oil-Lubricated Glass Substrates 

Glass slides were pre-treated (degreased and hydrophilized) with the plasma unit EQ-PDC-326 manufactured by MTI Co. (Berkley.CA, USA), equipped with a dry vacuum pump and pressure gauge EDWARDS 655AB (Burgess Hill, UK). Glass slides were exposed to an inductive air plasma discharge under the following parameters: the plasma frequency was 13.56 MHz; the pressure was 1 Torr; and the supplied power of plasma discharge was 18 W. The time span of irradiation was 120 s. Apparent contact angle of water established on plasma cleansed galls slides was 3–5°, evidencing complete wetting of slides with water and their satisfactory decontamination. Silicone oil (PDMS) was uniformly spread on the hydrophilized slide glass and formed a layer with a thickness of 3.8 ± 0.1 μm, as established by weighting. The uniformity of oil spreading was checked with optical microscopy. 

#### 2.2.2. Breath-Figures Self-Assembly under Drop-Casting of Polymer Solutions onto Silicone Oil-Lubricated Substrates 

**Figure 1:**
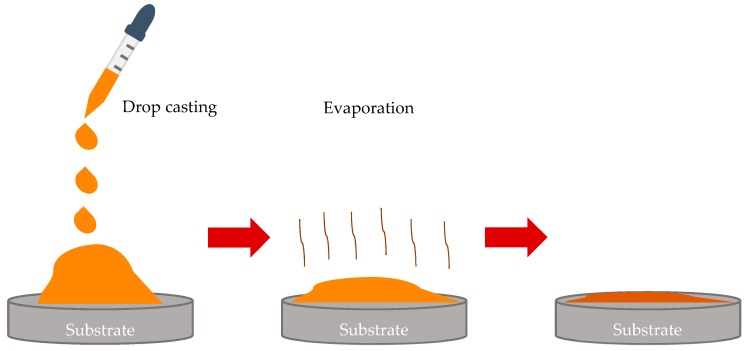
Sketch of the drop-casting process is depicted.

The prepared polymer solution, as described in Section 2.1., was casted by dropping onto the silicone oil-lubricated glass slide, as shown in Figure 1. The volume of the droplet was 30 µL. The process of drop-casting was carried under a temperature of 25 °C and relative humidity RH = 40%.

#### 2.2.3. Characterization of the Topography of the Patterns

The topography of the samples was studied with the SWIFT M4000D optical microscope (Schertz, TX, USA) and ultra-high resolution MAIA3 FE-SEM device (TESCAN, Brno, Czech Republic)). SEM images were processed with the software SEM Image Porosity and Pore Size for MATLAB, enabling extraction of porosity and pore size distribution from SEM images [33]. The ordering of the pores was quantified with Voronoi tessellations performed with the moduli of the MATLAB program developed at the Department of Physics and Astronomy at the University of California 

#### 2.2.4. Characterization of the Wetting Mode of the Breath-Figures Honeycomb Patterns 

Apparent contact angles were measured with the goniometer Rame-Hart 500 (Succasunna, NJ, USA) under ambient conditions. 

## 3. Results and Discussion

### 3.1. Breath-Figures Patterning on the Silicone Oil-Lubricated Solid Substrates

**Figure 2:**
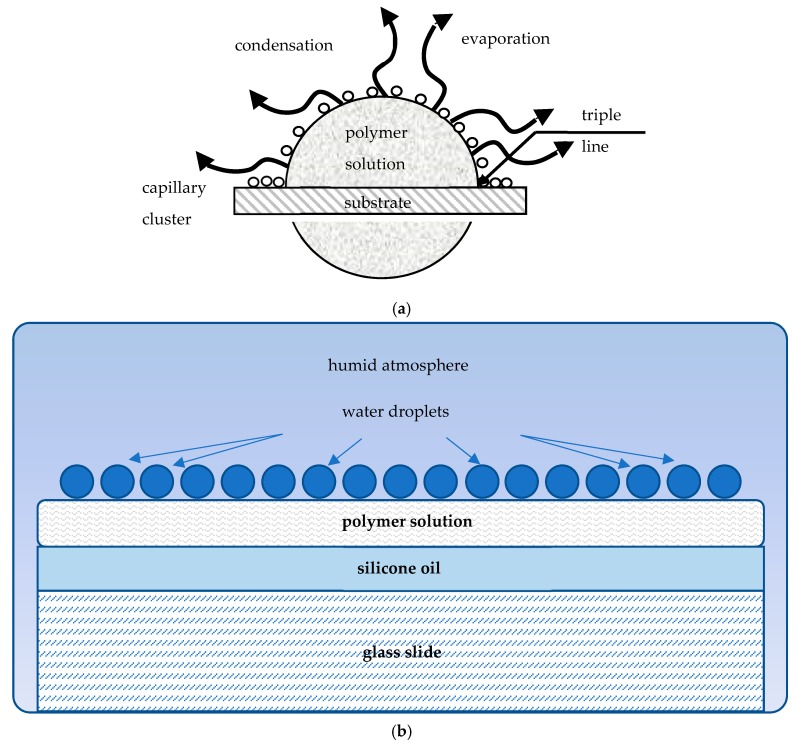
(**a**) Breath-figures self-assembly taking place under drop-casting is depicted. A droplet of the polymer solution was evaporated in the humid atmosphere. Water droplets were condensed at the polymer solution/vapor interface. The capillary cluster built of water droplets was formed in the vicinity of the triple (three-phase) line. (**b**) Breath-figures self-assembly taking place under drop-casting on silicone oil-lubricated surfaces is shown. Silicone oil promotes spreading of the polymer solution evaporated in the humid atmosphere.

The drop-casting involves a diversity of physicochemical events, namely: 1) spreading of droplet; 2) evaporation of the polymer solution; 3) nucleation and condensation of water droplets; 4) growth and self-assembly of droplets; 5) evaporation of water; 6) solidification of polymer accompanied, giving rise to the eventual microporous patterns, as shown in Figure 1 and discussed in detail in ref. [25]. The capillary cluster that gave rise to the formation of the eventual breath-figures patterns was formed in the vicinity of the triple (three-phase) line, as shown in Figure 2a. 

It has been reported that the substrate has a crucial impact on the breath-figures self-assembly [17,34,35,36]. In particular, it has been suggested that thick solid substrates (with a thickness of *ca* 1 mm), working at a thermal bath, stabilizes the process of evaporation of polymer solution [37]. In our research, we have modified the wetting regime of the substrate by lubricating with the silicone oil. The wetting of lubricated solid surface is rich in its physical content [38,39,40,41,42,43]. Silicone oils are usually (but not necessarily) used for lubrication. When a liquid wets a surface lubricated with another liquid, different wetting regimes are possible [38,39,40,41,42]. Consider a high surface energy droplet (say, water) placed on a low surface energy liquid (e.g., silicone oil)-lubricated surface. The oil may spread over and “cloak” the water droplet [38,39,40,41,42]. This is important because cloaking can stop the evaporation necessary for the breath-figures self-assembly. The criterion for coating (“cloaking”) is given by Equation (1):

(1) $$\Psi =\gamma_{wa}-\left(\gamma_{ow}+\gamma_{oa}\right)\;\;\;>0$$

where Ψ is the spreading coefficient; $\gamma_{wa}$, $\gamma_{wo}$ and $\gamma_{oa}$ are the surface tensions at the water/air, oil/water and oil/air interfaces, respectively [43,44]. In the situation where Ψ<0, the “non-coating” wetting regime takes place enabling the breath-figures self-assembly, and this was the case in our experiments, due to the relatively low surface tension of the polymer solution.

When droplets of the polymer solution were placed on the glass slide and silicone oil-lubricated glass slide, they behaved in very different ways, as illustrated in Figure 2a,b. When a droplet was placed on the dry slide glass, the triple line was pinned, as shown in Figure 2a. Contrastingly, when it was dripped on the lubricated slide, the triple line was de-pinned; the droplet spread and formed the so-called “puddle” with a thickness of 70 ± 10 μm, as depicted in Figure 2b. 

**Figure 3:**
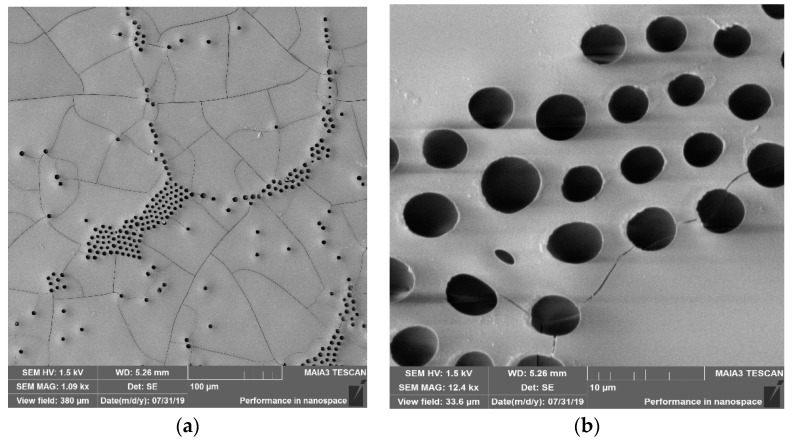
SEM images of breath-figures patterns obtained with drop-casting on the non-lubricated substrates are presented. (**a**) Scale bar is 100 µm; (**b**) scale bar is 10 µm.

The change of the wetting regime resulted in the dramatic change of eventual patterns obtained with “dry” and silicone oil-lubricated substrates. The patterns arouse from the drop-casting on dry glass slides are shown in Figure 3. The large-scale pattern with a characteristic lateral scale of 50 µm was recognized in the SEM image, supplied in Figure 3a. The origin of this pattern has remained highly disputable; it has been related to the crack patterns [45] and also to the diverse instabilities occurring in the evaporated polymer solution [46,47,48,49,50,51,52]. In parallel, the honeycomb microporous patterns, typical for the breath-figures self-assembly, were observed, as shown in Figure 3b. The pores constituting the pattern were disordered and demonstrated high dispersion of their sizes. 

**Figure 4:**
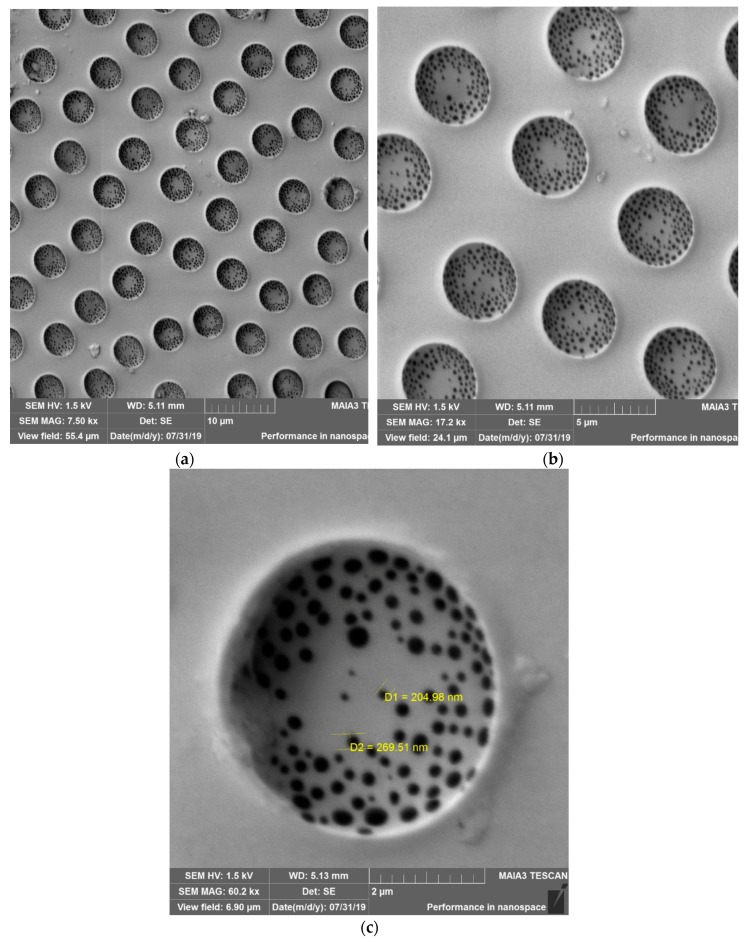
(**a**) SEM image of the breath-figures pattern obtained with silicone oil-lubricated substrates. The solution of PS with molecular mass $M_{w}=35,000$ was used for the breath-figures self-assembly. The scale bar is 10 µm; (**b**) large-scale SEM image of the breath-figures pattern obtained with silicone oil-lubricated substrates is shown. The scale bar is 5 µm; (**c**) the large-scale SEM image of the pore is depicted. The scale bar is 2 µm. Nanopores are clearly visible.

**Figure 5:**
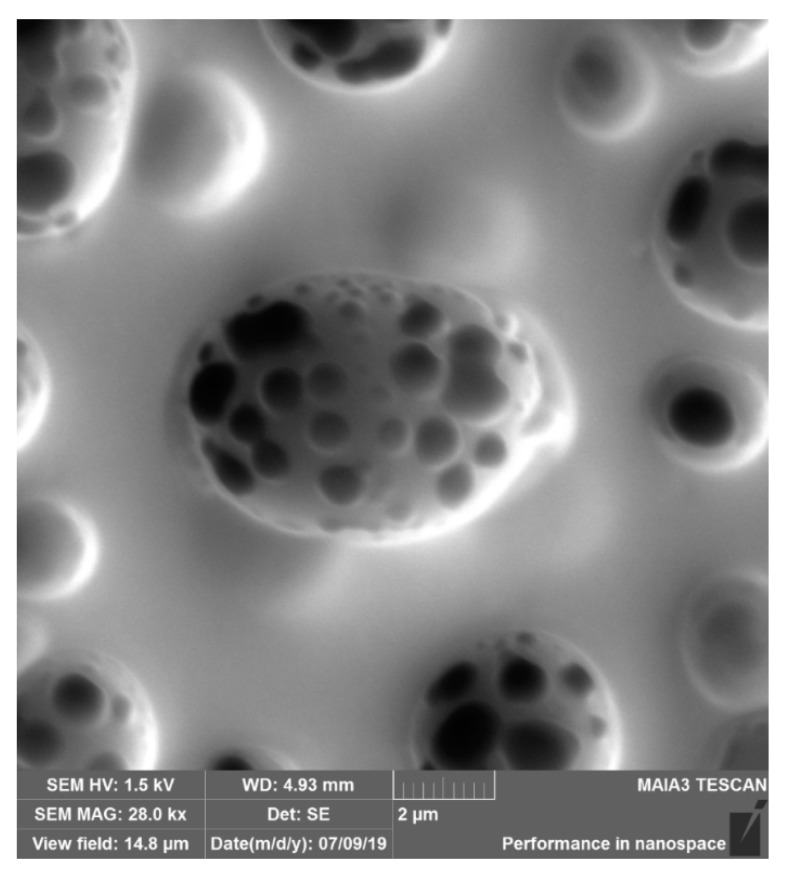
SEM image of the breath-figures pattern obtained with silicone oil-lubricated substrates is depicted. The solution of industrial PS was used for the breath-figures self-assembly. The scale bar is 2 µm.

**Figure 6:**
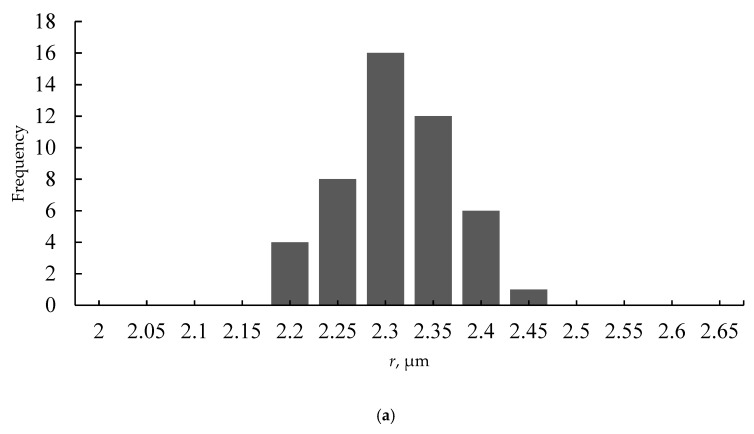

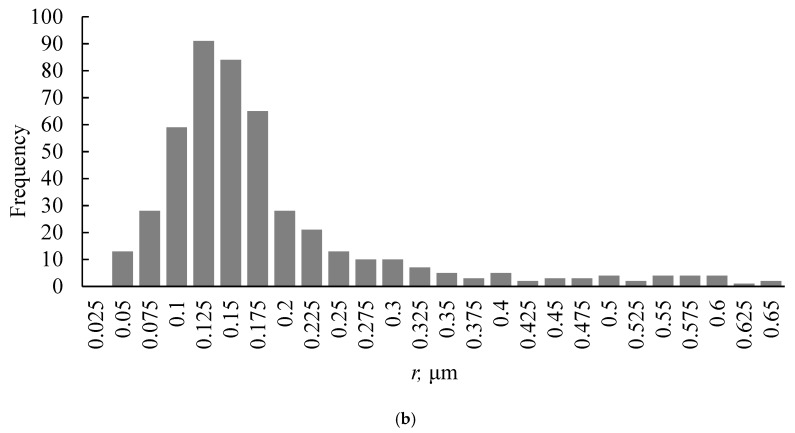
Pores size (radius) distribution is depicted. Typical numbers of pores possessing diameters confined within a given range appearing on the SEM images are supplied. (**a**) Large micropores size distribution extracted from SEM images is shown; (**b**) size distribution of nanopores is shown.

The situation changed dramatically when the polymer solution was deposited on the silicone oil-lubricated substrates, as shown in Figure 4a–c. The aforementioned large-scale pattern disappeared. The honeycomb microporous ordered pattern, depicted in Figure 4a–c, was observed. It should be emphasized that the pattern was hierarchical, namely small-scale nanopores were located within “large” micro-scaled pores, as shown in Figure 4b,c. The diameters of the nanopores were established with SEM as 100–300 nm. The presence of nano-scaled pores makes the reported films suitable for ultra-filtration. The formation of hierarchical porous structures under the breath-figures self-assembly has been addressed in Reference [12]. However, the precise mechanism of their formation remains obscure and calls for future investigations. Hierarchical porous structures were registered with both PS ($M_{w}=35,000\frac{g}{mol})$ and the industrial grade PS, as shown in Figure 5. The size distribution of micro and nanopores, extracted from SEM images, is shown in Figure 6. The typical radius of micropores was established as 2.3 µm; in turn the typical diameter of nanopores was 125 nm. 

The irregular shapes of the pores, depicted in Figure 5, are typical for the patterns obtained with industrial grade PS. It is reasonable to relate this observation to the broad molecular mass distribution inherent in industrial grade polymers. It is a well-known fact that the molecular mass of polymer exerts a decisive impact on the breath-figures patterns [14,15,16,17]. However, the precise mechanism of this impact remains unclear to a large extent. 

### 3.2. Mechanism of the Formation of the Hierarchical Patterns

**Figure 7:**
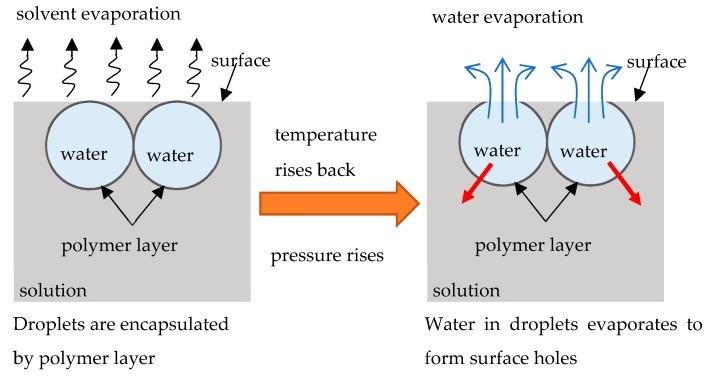
The mechanism of pores formation, assuming the bursting “hypothesis”, suggested in Reference [53] is depicted. Red arrows indicate formation of nanopores, under disruption of the polymer shell.

A reasonable question to be addressed is: what is the physical mechanism of the formation of the small-scale patterns, built of nanopores, shown in Figure 4b,c. Obviously, the formation of these pores could not be explained with the traditional “condensation”-inspired mechanism, introduced by investigators [14,15,16,17]. Perhaps, the formation of the nanopores obtains the explanation within the so-called “bursting hypothesis” introduced in Reference [53]. Ma et al. suggested [53] that at the early stage of the breath-figures process, the solvent is evaporated so fast that the temperature of the solution drops enough preventing the evaporation of water droplets. As a result, the polymer layer encapsulating the droplets is formed, as shown in Figure 7. After this, the temperature rises, the pressure within the micropores filled with water is increased, the polymer shell is burst and water is evaporated [53]. It is plausible to suggest that, at this stage, when the pressure within pores is increased, the water vapor disrupts the polymer shell encapsulating droplets in numerous points; thus, resulting in the formation of the nanopores, as shown in Figure 4b,c. The “bursting hypotheses” explains numerous experimental observations, reviewed in References [14,15,16,17]; however, this speculation of course calls for additional experimental justification. 

### 3.3. Characterization of Ordering of the Breath-Figures Patterns

**Figure 8:**
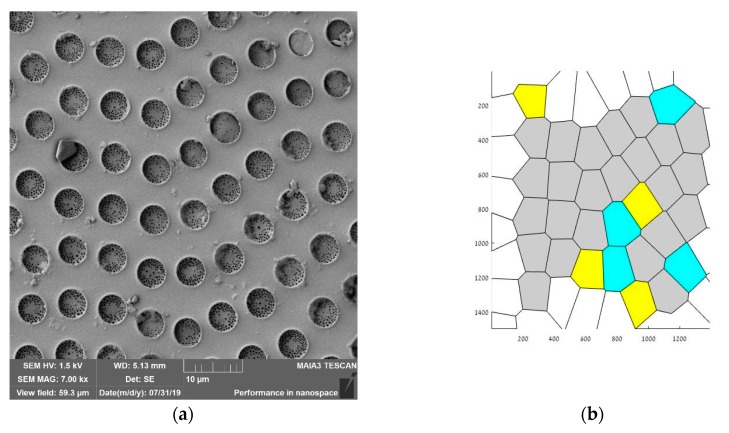
The SEM image of the honeycomb pattern (**a**) and the appropriate Voronoi tessellation (**b**) are shown. The calculated Voronoi entropy *S_vor_* =0.736. Grey polygons are hexagons, yellow polygons are pentagons and blue polygons are heptagons.

Ordering of the pores was quantified with the Voronoi tessellations method. A Voronoi tessellation or diagram of an infinite plane is a partitioning of the plane into regions based on the distance to a specified discrete set of points, also called the nuclei (or generators [54,55]). For each seed, there is a corresponding region consisting of all the points closer to that seed than to any other. The centers of the large micro-scaled pores depicted in Figure 4a,b, were taken as nuclei (thus, the small-scale pores were ignored). The Voronoi entropy was calculated as Equation (2):

(2) $$S_{vor}=-\sum_{n}P_{n}\ln P_{n}$$

where *P_n_* is the fraction of polygons with *n* sides or edges (also called the coordination number of the polygon) in a given Voronoi diagram [54,55]. Voronoi entropy was successfully used for characterization of ordering in breath-figures patterns in References [55,56,57,58,59]. The values of Voronoi entropy $S_{vor}=0.41-0.48$ were reported in Reference [10] for the breath-figures patterning, which are much lower than $S_{vor}=1.71$, inherent for the random distributions of pores [59]; thus, evidencing the pronounced ordering of micropores. We established $S_{vor}=0.6-0.9$ for the reported patterns (see Figure 8), also evidencing the ordering of pores. However, the observed ordering was far from to be comprehensive (consider $S_{vor}=0$ for the “ideal” ordering).

### 3.4. Characterization of Wetting of the Samples Arising from the Breath-Figures Self-Assembly

**Figure 9:**
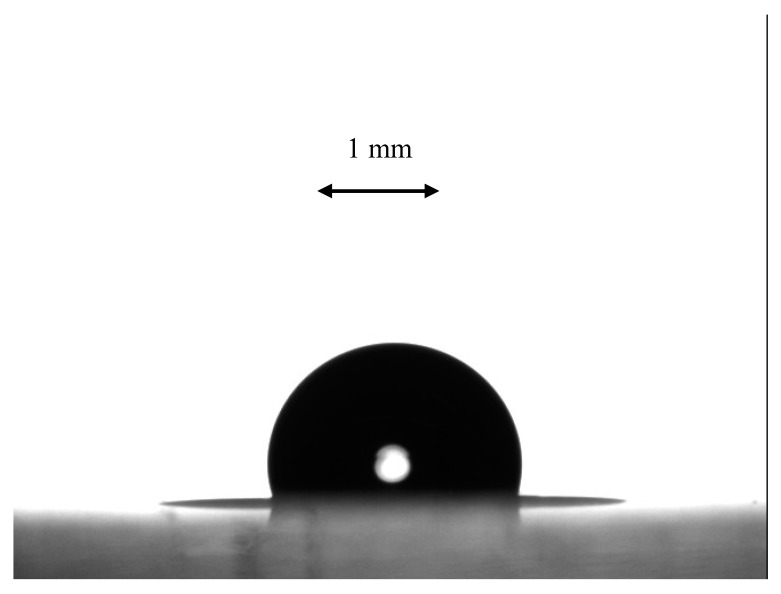
Obtuse apparent water contact angle measured on PS honeycomb surfaces, obtained under breath-figures self-assembly with oil-lubricated solid surfaces is shown. The volume of the water droplet is 5 µL. The scale bar is 1 mm.

Measurement of the apparent contact angles remains the simplest, inexpensive and reliable method of characterization of porous surfaces [41,42,60,61]. The equilibrium contact angle of flat PS was established as *θ =* 86–88° [62]. The apparent contact angle of the porous topographies arising from the breath-figures self-assembly on oil-lubricated surfaces was established as θ^*^ = 100–107 ± 1° as shown in Figure 9. Thus, we conclude that the Cassie-Baxter air trapping regime is inherent for the reported patterns [43,44]. Consider that under the Wenzel wetting regime, an inherently hydrophilic substrate becomes more hydrophilic. Of course, the Cassie and Wenzel wetting models do not exhaust all possible wetting regimes; more complicated wetting regimes are widespread on real surfaces, and the so called “mixed” wetting mode is possible for the reported patterns [43].

## 4. Conclusions

We conclude that the silicone oil-lubricated substrates promote de-pinning of the triple line of droplets of polymer solutions and their spreading; thus, these substrates are especially suitable for the drop-casting process, resulting in the breath-figures self-assembly [12,13,14,15,16,17]. The drop-casting of polystyrene dissolved in the chlorinated solvents on the silicone oil-lubricated glass slides, carried out in a humid atmosphere, gave rise to hierarchical topographies built of micro and nanopores. The size distribution of pores was reported. The typical diameter of the nanopores was established as 125 nm. The hierarchical topography was observed with industrial grade polystyrene and polystyrene with a molecular weight of $M_{w}=35,000\frac{g}{mol}$. The mechanism of formation of the hierarchical patterns was suggested. The presence of nanopores makes the reported films suitable for ultra-filtration applications. The ordering of the micropores was quantified with the Voronoi tessellations and calculation of the corresponding Voronoi entropy. The Voronoi entropy was established as $S_{vor}=0.6-0.9$, which is much smaller than $S_{vor}=1.71$ inherent in random 2D patterns [54,55,56,57,58,59]. Obtuse apparent contact angles established for the reported honeycomb films evidence the Cassie-Baxter air trapping wetting regime.
